# Computational Identification of BCR-ABL Oncogenic Signaling as a Candidate Target of Withaferin A and Withanone

**DOI:** 10.3390/biom12020212

**Published:** 2022-01-26

**Authors:** Vidhi Malik, Navaneethan Radhakrishnan, Sunil C. Kaul, Renu Wadhwa, Durai Sundar

**Affiliations:** 1 DAILAB, Department of Biochemical Engineering & Biotechnology, Indian Institute of Technology (IIT)-Delhi, Hauz Khas, New Delhi 110-016, India; vidhi0205@gmail.com (V.M.); navaneethan@dbeb.iitd.ac.in (N.R.); 2 AIST-INDIA DAILAB, DBT-AIST International Center for Translational & Environmental Research (DAICENTER), National Institute of Advanced Industrial Science & Technology (AIST), Tsukuba 305-8565, Japan; s-kaul@aist.go.jp (S.C.K.); renu-wadhwa@aist.go.jp (R.W.); 3 School of Artificial Intelligence, Indian Institute of Technology (IIT) Delhi, New Delhi 110-016, India

**Keywords:** BCR-ABL, leukemia, Philadelphia chromosome, Ashwagandha, Withaferin A, Withanone, CML therapy

## Abstract

Withaferin-A (Wi-A), a secondary metabolite extracted from Ashwagandha (*Withania somnifera*), has been shown to possess anticancer activity. However, the molecular mechanism of its action and the signaling pathways have not yet been fully explored. We performed an inverse virtual screening to investigate its binding potential to the catalytic site of protein kinases and identified ABL as a strong candidate. Molecular docking and molecular dynamics simulations were undertaken to investigate the effects on BCR-ABL oncogenic signaling that is constitutively activated yielding uncontrolled proliferation and inhibition of apoptosis in Chronic Myeloid Leukemia (CML). We found that Wi-A and its closely related withanolide, Withanone (Wi-N), interact at both catalytic and allosteric sites of the ABL. The calculated binding energies were higher in the case of Wi-A at catalytic site (−82.19 ± 5.48) and allosteric site (−67.00 ± 4.96) as compared to the clinically used drugs Imatinib (−78.11 ± 5.21) and Asciminib (−54.00 ± 6.45) respectively. Wi-N had a lesser binding energy (−42.11 ± 10.57) compared to Asciminib at the allosteric site. The interaction and conformational changes, subjected to ligand interaction, were found to be similar to the drugs Imatinib and Asciminib. The data suggested that Ashwagandha extracts containing withanolides, Wi-A and Wi-N may serve as natural drugs for the treatment of CML. Inhibition of ABL is suggested as one of the contributing factors of anti-cancer activity of Wi-A and Wi-N, warranting further in vitro and in vivo experiments.

## 1. Introduction

Cancer is most consistently defined by uncontrolled division of cells yielding their abnormal mass at any local site in the body or even spread to distant organs through the blood or lymphatic stream by a process called metastasis [1]. Chronic myelogenous leukemia (CML) is a type of cancer in which the bone marrow produces an excess of white blood cells of myelogenous origin. Most prevalent in middle-aged individuals, it often complicates into infections, anemia, and bleeding. Accounting for about 15% of all cases of leukemia, CML is clinically categorized as chronic, accelerated and blastic, with <10%, 10–20% and >20% blast cells in the blood/ bone marrow, respectively [2]. Since the cancerous cells in CML are disposed directly into the bloodstream, such a disease has a higher incidence of multiple organ involvement and is often associated with poor prognosis.

Pathogenesis of CML involves the non-hereditary formation of the Philadelphia Chromosome [3]. The latter is characterized by chromosome 9–22 translocation/t(9;22)(q34;q11) yielding BCR-ABL fusion protein-the classical proteomic hallmark of the disease. BCR has serine/threonine kinase activity harboring ABL-interactive Rho-GEF/Rac-GAP domains [4,5]. It is considered to be a cytoplasmic protein; however, it also has the ability to bind to membrane proteins through its pleckstrin homology (PH), Ca^2+^-dependent phospholipid binding C2 domains and PDZ-domain-binding C-terminal sequence [5]. The PDZ-domain-binding C-terminus helps it to control cell proliferation by Ras-mediated signaling via interaction with AF-6 junctional PDZ-domain proteins present on the surface of the epithelial cells [5,6]. On the other hand, BCR-interactive SH3/SH2 domain harboring ABL1 protein regulates proliferation, differentiation, cytoskeletal reorganization, survival or apoptosis and migration of cells in response to growth factors, cytokines, oxidative stress and DNA damage [7,8]. Thus, the BCR-ABL fusion protein houses the ability to promote cell proliferation and prevent apoptosis [9,10]. Activities of the ABL protein are tightly regulated by its amino terminal SH2 and SH3 domains. In the normal cells, ABL exists in an ‘auto-inhibited’ state via interaction of its myristate (Myr)-bound cap with allosteric site at the base of the catalytic domain [11]. Binding of myristic acid at the allosteric site induces a conformational change in its catalytic domain, causing disruption of αI helix at Phe516 resulting into a 90° turn, followed by another helical turn involving four residues (Phe516 to Ser519, = αI’ helix) [12]. This conformational change in turn causes the SH2 domain to interact with the catalytic domain in such a way that it prevents the interaction of phosphorylated tyrosine proteins with SH2 domains thereby inducing an inactive state of the protein [11]. The importance of Myr-binding was revealed by mutating the extreme amino terminal residue of the cap that led to an activation of ABL protein both in vitro and in vivo [13,14]. BCR-ABL fusion protein lacks Myr-bound cap region and thus stays in a constitutively active state, with enhanced stability and binding to F-actin, resulting in hyper-proliferation of the CML cells [11].

Conventional clinico-therapeutic options for CML are targeted therapy, biological therapy, high dose chemotherapy with a stem cell transplant, donor lymphocyte infusion (DLI), and supportive surgical interventions (splenectomy). Targeted therapy includes the use of tyrosine kinase inhibitors selective against the cancer cells. Imatinib mesylate (or Gleevec^TM^) is the catalytic inhibitor of BCR-ABL protein activity and is FDA-approved for treatment of CML [11]. Imatinib binds to the BCR-ABL tyrosine kinase ATP binding site and inactivates it by stabilizing its non-ATP-bound form [15]. However, BCR-ABL catalytic domain mutations driven-relapses have been commonly reported in patients treated with Imatinib [11]. Studying the mechanism of drug resistance may help in the development of better approaches targeting the BCR-ABL protein structure and/or function. Likewise, in recent studies, dual targeting of ABL protein via Asciminib (allosteric inhibitor) and Nilotinib/Axitinib (catalytic site inhibitor) was suggested as a potential and promising strategy meriting the non-overlapping drug resistance profiles of these inhibitors [16,17]. Several available drug regimens have been reported to result in severe adverse effects, drug resistance, and disease recurrence [17,18,19], urging for the investigation of new molecules or combination therapy with higher chemotherapeutic index, safety and affordability [20].

**Figure 1:**
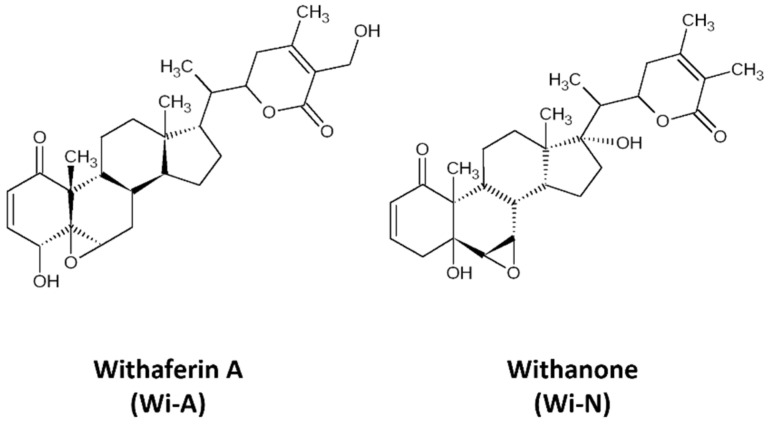
The 2D representations of structures of Withaferin A (Wi-A) and Withanone (Wi-N).

Withaferin-A (Wi-A), a secondary metabolite extracted from *Withania somnifera* (Ashwagandha), has been shown to possess anticancer activities [21,22,23,24,25]. Wi-A and its structural analog Withanone (Wi-N) has been well characterized from Ashwagandha by our group and has been studied for its anti-cancer activities for over a decade (Figure 1) [22,26,27,28,29,30,31,32]. These have been shown to target mortalin-p53, NEMO/IKKβ, TPX2-Aurora A, hnRNP-K and EGFR driven oncogenic signaling [22,26,29,33] yielding multimodal anti-cancer activity. Most of these studies investigated the interaction of Wi-A and Wi-N with specific targets. Here, we undertook an unbiased approach and explored their possible targets using inverse virtual screening on the catalytic site of protein kinases. We identified ABL as the top scorer. Molecular docking and molecular dynamics simulations were then performed to check the potential of Wi-A and Wi-N, as the catalytic and allosteric inhibitors of ABL protein. Imatinib and Asciminib were taken as controls for catalytic and allosteric inhibition of ABL, respectively. In silico analyses showed that while Imatinib acts at the catalytic site and Asciminib acts at the allosteric site, Wi-A could mimic the action of both, endorsing its potential as catalytic as well as allosteric inhibitor of ABL protein. Wi-N, on the other hand interacted with the allosteric site. We also investigated the effect of the combination of Wi-A and Wi-N and report that they have considerable potential to cause catalytic and allosteric inhibition of ABL, and hence warrant laboratory and clinical studies.

## 2. Materials and Methods

### 2.1. Inverse Virtual Screening of Wi-A as Inhibitor of Protein Kinases

Wi-A was screened for 851 kinase protein-ligand structures downloaded from scPDB database [34], using xglide.py script of Schrodinger suite 2018-2 including standard precision (SP) algorithm of Glide [35].

### 2.2. Molecular Docking and Simulation of Wi-A and Wi-N at Catalytic and Allosteric Site of ABL Protein

ABL was identified as the top scoring target of Wi-A from inverse virtual screening experiment. In silico experiments to efficiently target ABL protein with Wi-A and its less toxic structural analog, Wi-N, was designed. Imatinib and Asciminib were used as controls for catalytic and allosteric site inhibitors, respectively. The method used here is similar to our previously published study that can be referred for more details [36].

#### 2.2.1. Preparation of Structures

Crystal structure of ABL1 was obtained from Protein Data Bank (PDB ID: 1OPL) [13,14]. The structures of ligands, Imatinib, Asciminib, Wi-A and Wi-N, were retrieved from PubChem database [37], and prepared using Schrodinger 2019-2 modules, PrepWizard and LigPrep, respectively. To the crystal structure of the protein, hydrogens were added, termini were capped, missing side chains were added and protonation states were assigned. A restrained energy minimization was done using OPLS3 forcefield in which the hydrogen atoms were energy minimized. Tautomeric states, stereoisomers and ionization states corresponding to pH 7 were generated for the drug molecules.

#### 2.2.2. Molecular Docking and MD Simulations to Check the Potential of Natural Compounds as Inhibitors of ABL

Both catalytic and allosteric sites of ABL protein were targeted to check the inhibitory effects of Wi-A and Wi-N, by molecular docking and molecular dynamics simulation studies. Glide was used to perform docking of ligands at the catalytic and allosteric sites of protein [35,38]. Docking grids of dimensions 10 Å were generated around the catalytic and allosteric sites and extra precision (XP) algorithm with flexible ligand sampling was used. Scaling factor for van der Waals radii was set to 0.80 and the remaining options were set to default. The docked complexes were simulated to monitor the stability of the ligand bound complexes and conformational changes induced by them using Desmond module of Schrodinger suite [35]. The protein-ligand complexes were simulated using Optimized Potential for Liquid Simulations 3 (OPLS3) force field in a TIP4P solvated periodic box with 10 Å spacing. The solvation of the complexes was followed by neutralization and minimization for up to 2000 iterations. Minimized systems were equilibrated in seven steps in NVT and NPT ensembles using the relaxation protocol defined in Desmond Schrodinger suite [35]. The production simulations were performed for 50 ns in NPT ensemble with a time-step of 2 fs. Temperature was maintained at 300 K using Nose–Hoover chain thermostat with a relaxation time of 1 ps and pressure was maintained at 1 atm using Martyna–Tobias–Klein barostat with a relaxation time of 2 ps. The cutoff radius for short range Coulombic interactions was set to 9 Å. No restraints were applied to any molecule. All other options were set to default.

#### 2.2.3. Analysis of MD Simulated Systems

Root mean square deviation (RMSD), hydrogen bonds analysis and conformational changes over the simulation trajectories of protein-ligand complexes were monitored using VMD version 1.9.4 [39]. The three-dimensional representations presented in this study were generated using PyMol molecular graphics system [40]. The two-dimensional representations were generated using Schrodinger suite. The MM/GBSA (molecular mechanics energies combined with the generalized Born and surface area continuum solvation) free energies of binding of protein-ligand complexes before and after simulations were calculated using the Prime module of the Schrodinger suite 2019-2. Prime MM/GBSA uses VSGB 2.0 energy model and the equation used in the calculation is:

MM/GBSA ΔG_bind_ = ΔG_complex_ − (ΔG_receptor_ + ΔG_ligand_)

ΔG_complex_, ΔG_receptor_ and ΔG_ligand_ represent the free energies of the complex, receptor and ligand, respectively. The details of MM/GBSA binding energy calculation is reported elsewhere [41,42]. Binding energies were calculated for 40 snapshots that were saved every 100 ps from 11–50 ns of simulation trajectories. The average values with their standard deviation have been reported. The protein ligand interactions during last 40 ns of simulation were visualized using ‘Simulation Interactions Diagram’ module in Schrodinger suite.

Videos of the simulation trajectories have been included as Supplementary Files S2–S5.

#### 2.2.4. Cytotoxicity Assay

Cytotoxicity of Wi-A and Wi-N was determined by quantitative cell viability assay using MTT [3-(4,5-dimethylthiazol-2-yl)-2,5-diphenyltetrazolium bromide] assay (Sigma Aldrich, Tokyo, Japan) using human cancer cells including breast carcinoma (MCF-7, T47D), colorectal carcinoma (DLD1 and HCT116), cervical carcinoma (HeLa), osteosarcoma (U2OS), esophageal carcinoma (T.Tn) and myelogenous leukemia (K562). Cells were seeded in 96-well plates at a density of 5 × 10^3^ cells/well and incubated for 24 h at 37 °C in a CO_2_ incubator. The cells were treated with Wi-A (0.25, 0.50, 1.0, 1.50, 2.0 and 3.0 μM) or Wi-N (5, 10, 20, 40 and 60 μM) supplemented medium (DMEM) for 48 h, followed by the addition of MTT solution (0.5 mg/mL; 100 μL/well) and incubation at 37 °C for further 3–4 h. DMSO (100 μL) was then added and the plates were shaken for 10 min for dissolution of formazan crystals. The absorbance was measured at 570 nm using Infinite M200 Pro microplate reader (Tecan Group Ltd., Männedorf, Switzerland).

## 3. Results

### 3.1. Identification of ABL Protein as a Top Target for Wi-A by Inverse Virtual Screening

**Figure 2:**
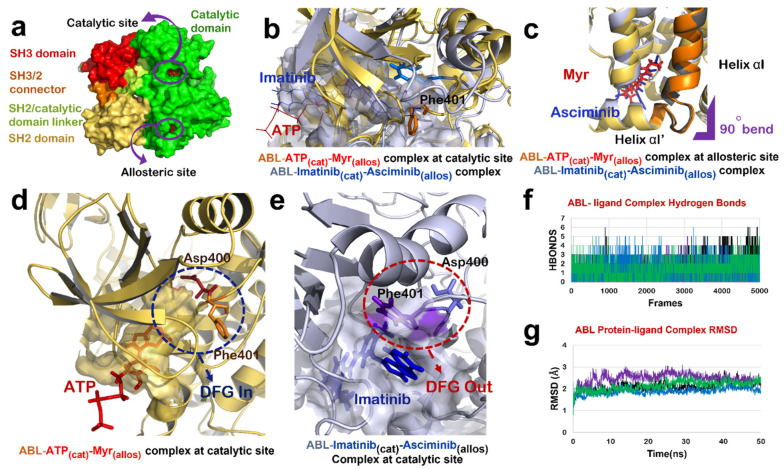
ABL-Imatinib_(cat)_-Asciminib_(allos)_ Complex comparison with ABL-ATP-Myr complex. (**a**) Different domains of ABL protein in chain-A of 1OPL PDB structure; catalytic domain (green), SH3/2 connector (orange), SH2 domain (yellow) and SH3 domain (red). (**b**) Superimposed structure of catalytic site of ABL-ATP-Myr complex (ABL in yellow, ATP in red) and ABL-Imatinib_(cat)_-Asciminib_(allos)_ (ABL in grey, Imatinib in blue). (**c**) Superimposed structure of allosteric site of ABL-ATP-Myr complex (ABL in yellow, Myr in red) and ABL-Imatinib_(cat)_-Asciminib_(allos)_ complex (ABL in grey, Asciminib in blue), 90◦ bend highlighted by purple mark is induced by both Myr and Asciminib followed by formation of 4 residues αI’ helix. (**d**) DFG-‘in’ conformation of motif as induced by binding of natural substrate, ATP, at the catalytic site of ABL protein. (**e**) Molecular action mechanism of change in conformation of DFG motif induced by Imatinib at the catalytic site of ABL protein. (**f**) Hydrogen bonds plot of number of interactions formed by protein-ligand complexes, ABL-Imatinib_(cat)_-Asciminib_(allos)_ complex (black), ABL-Wi-A_(cat)_-Wi-N_(allos)_ complex (purple), ABL-Wi-A_(cat)_-Wi-A_(allos)_ complex (blue) and ABL-Imatinib_(cat)_-Wi-A_(allos)_ (green). (**g**) RMSD plot of protein-ligand complexes, ABL-Imatinib_(cat)_-Asciminib_(allos)_ complex (black), ABL-Wi-A_(cat)_-Wi-N_(allos)_ complex (purple), ABL-Wi-A_(cat)_-Wi-A_(allos)_ complex (blue) and ABL-Imatinib_(cat)_-Wi-A_(allos)_ (green).

**Figure 3:**
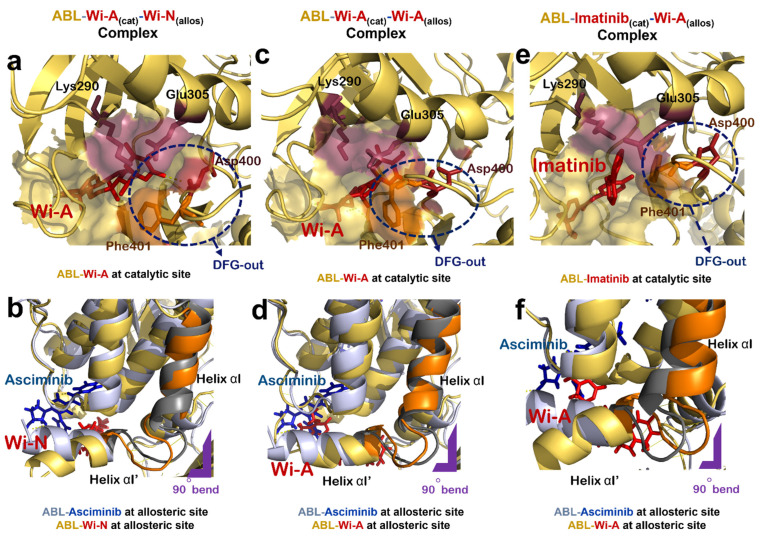
Effect of natural compounds on catalytic and allosteric site of ABL protein dimer. (**a**) Interaction of Wi-A at catalytic site of ABL-Wi-A_(cat)_-Wi-N_(allos)_ complex (ABL in yellow, Wi-A in red). (**b**) Superimposed structure of allosteric site of ABL-Imatinib_(cat)_-Asciminib_(allos)_ complex (ABL in grey, Asciminib in blue) with that of ABL-Wi-A_(cat)_-Wi-N_(allos)_ complex (ABL in yellow, Wi-N in red). (**c**) Interaction of Wi-A at catalytic site of of ABL-Wi-A_(cat)_-Wi-A_(allos)_ complex (ABL in yellow, Wi-A in red). (**d**) Superimposed structure of allosteric site of ABL-Imatinib_(cat)_-Asciminib_(allos)_ complex (ABL in grey, Asciminib in blue) with that of ABL-Wi-A_(cat)_-Wi-A_(allos)_ complex (ABL in yellow, Wi-A in red). (**e**) Interaction of Imatinib at catalytic site of ABL-Imatinib_(cat)_-Wi-A_(allos)_ complex (ABL in yellow, Imatinib in red). (**f**) Superimposed structure of allosteric site of ABL-Imatinib_(cat)_-Asciminib_(allos)_ complex (ABL in grey, Asciminib in blue) with that of Abl-Imatinib_(cat)_-Wi-A_(allos)_ complex (ABL in yellow, Wi-A in red).

Considering the role of constitutively activated kinases in oncogenic signaling, and anticancer activity of Wi-A demonstrated in previous studies, we anticipated that it may inhibit kinases and hence performed inverse virtual screening using the catalytic site of protein kinases. ABL proto-oncogene 1, AKT serine/threonine kinase 1, BRAF were identified as top candidate target proteins. Among the list (Supplementary Table S1), ABL1 showed the top score for binding of Wi-A at its catalytic site. We next designed further in silico experiments to investigate the docking potential of Wi-A and Wi-N to the catalytic and allosteric sites of ABL protein. Imatinib and Asciminib were used as controls for targeting the catalytic and allosteric sites of ABL, respectively. The potential of Wi-A and Wi-N to serve as catalytic or allosteric inhibitor was analyzed by docking these compounds at catalytic and allosteric sites of ABL protein (Table 1). The binding energies of Wi-A and Wi-N were compared with the binding energies of Imatinib at catalytic site and Asciminib at allosteric site. This initial screening of natural compounds showed us that Wi-A could act as both catalytic and allosteric inhibitor, showing binding energies of −6.89 and −3.58 kCal/mol, respectively. On the other hand, Wi-N could serve only as allosteric inhibitor with a binding energy of −2.67 kCal/mol (Table 1). The preliminary screening results set a foundation for designing further in silico experiments for dual targeting of ABL at both catalytic and allosteric sites by docking the combinations of inhibitors at catalytic and allosteric sites to generate four complexes, ABL-Imatinib_(cat)_-Asciminib_(allos)_ (treated as positive control), ABL-Wi-A_(cat)_-Wi-A_(allos)_, ABL-Wi-A_(cat)_-Wi-N_(allos)_, and ABL-Imatinib_(cat)_-Wi-A_(allos)_ complexes. The docking scores, binding energies calculated during simulations and the interacting residues of ABL are shown in Table 2. The 3D and 2D representations of interactions are shown in Figure 2 and Figure 3, and Supplementary File S1: Figure S1 and Figure S3 respectively. Supplementary File S1: Figures S2 and S3 show the fraction of simulation time during which specific interactions were maintained between ABL residues and the inhibitors.

### 3.2. Dual Targeting of ABL with Imatinib and Asciminib

Mechanism of interaction of both Imatinib and Asciminib with ABL protein has been revealed by crystallographic studies [16,43]. It has been shown that the Imatinib binds to the ATP-binding site of ABL and inhibits its activity by restricting the binding of ATP and stabilizing the inactive conformation of protein, whereas Asciminib interacts at the Myr-binding site to induce the formation of αI’ helix with a 90° bend as observed in the case of binding of its natural substrate (Myr) [16]. We studied the effect of combination of Imatinib and Asciminib (as its allosteric inhibitor) on ABL protein structure (Figure 2a). Molecular docking and dynamic studies revealed that Imatinib binds to the ATP binding site (Figure 2b,e) and Asciminib interacts at the Myr-binding site to induce the formation of αI’ helix with a 90° bend as reported in a crystallographic study [16] (Figure 2c). Imatinib induced an ‘out’ conformation of its DFG motif in the activation loop in which Asp points in an outward direction of the ATP binding site while Phe points towards it (Figure 2b,e). In the active state of ABL protein, these two residues reside in the reverse orientation thereby favoring ‘DFG-in’ conformation of this motif (Figure 2b,d). ABL-Imatinib_(cat)_-Asciminib_(allos)_ complex was simulated for 50 ns, and the structure was stable over the period of simulation (Figure 2f,g). BCR-ABL fusion protein does not have a cap region, due to which Myr cannot bind to its binding site to keep ABL in an inactive state. The data implied that a combination of Imatinib and Asciminib could be considered as a therapy for targeting BCR-ABL fusion protein lacking the Myr-bound cap, as Asciminib can induce an inactive conformation of this fusion protein the way it was naturally induced by Myr-binding. Additionally, Imatinib may act as competitive inhibitor of ABL, thereby preventing the binding of ATP at its binding site and inducing an inactive conformation of its DFG motif.

### 3.3. Dual Targeting of ABL with Wi-A as Catalytic Inhibitor and Wi-N as Allosteric Inhibitor

Next, the docking potential of combination of Wi-A and Wi-N on ABL protein was examined in a similar manner. Wi-A showed a good binding affinity of −75.87 ± 4.75 kCal/mol at the catalytic site of ABL, whereas Wi-N bound to the allosteric site with binding energy of −42.11 ± 10.57 kCal/mol (Table 2). The ABL-Wi-A_(cat)_-Wi-N_(allos)_ complex was simulated for 50 ns and it was observed that the complex got stable within initial 10 ns of simulation (Figure 2g). Molecular interaction studies at the catalytic site of protein showed that Wi-A stably interacted at catalytic site and formed majority of interactions with Imatinib interacting residues Leu267, Gly268, Tyr272, Lys290, Phe336, Thr338, Leu389 and Phe401 (Table 2 and Supplementary File S1: Figures S1a, S1e, S2a and S2e). Also, the ionic pair between Lys290 and Glu305 was intact and the DFG-‘out’ conformation was induced by Wi-A at catalytic site of ABL protein (Figure 3a). At the allosteric site, the number of interacting residues and binding energy of Wi-N was comparatively lower than that of Asciminib (Table 2 and Supplementary File S1: Figures S1b, S1f, S2b and S2f). Wi-N did not show interaction with Asciminib interacting residues like Ala356, Ala452 and Glu481 (Supplementary File S1: Figure S2b,f). However, Wi-N interacted with allosteric site residues like Glu353, Val357 and Asn355. Interestingly, Wi-N was able to induce the conformational change at the allosteric site similar to the one induced by Asciminib and Myr (Figure 3b). Therefore, it was concluded that the combination of Wi-A and Wi-N might mimic the binding mechanism of Imatinib and Asciminib, respectively.

### 3.4. Dual Targeting of ABL with Wi-A as Both Catalytic and Allosteric Inhibitor

Wi-A showed a higher binding affinity at the allosteric site of ABL than Wi-N in our initial protein allosteric site inhibitor docking screening (Table 1). Therefore, we examined the effect of Wi-A at both catalytic and allosteric sites of protein by generating an ABL-Wi-A_(cat)_-Wi-A_(allos)_ complex (Figure 3c,d). Wi-A showed stronger binding at the allosteric site with a binding energy of −67.00 ± 4.96 kCal/mol as compared to the −42.11 ± 10.57 kCal/mol binding energy of Wi-N (Table 2). In case of ABL-Wi-A_(cat)_-Wi-A_(allos)_ complex, Wi-A showed stronger binding affinity at both the catalytic and allosteric site of ABL as compared to Imatinib and Asciminib and was able to form majority of interactions with Imatinib interacting residues-Thr338, Gly268 and Tyr272-at the catalytic site of ABL (Table 2 and Supplementary File S1: Figures S1c, S2c and S3c). Further, Wi-A was able to induce DFG-out conformation of DFG motif and the ionic pair between Lys290 and Glu305 was also intact at the catalytic site of ABL (Figure 3c). At the allosteric site of ABL, Wi-A stably interacted with some of Asciminib interacting residues like Ala356, Arg351, Asn355, Leu529 and Tyr454, and mimicked the conformational changes induced by Asciminib (Figure 3d and Supplementary File S1: Figures S1d, S2d and S3d). This data implied that Wi-A alone might have a potential to serve as both catalytic and allosteric inhibitor of ABL protein activity.

### 3.5. Dual Targeting of ABL with Imatinib as Catalytic Inhibitor and Wi-A as Allosteric Inhibitor

The effect of combination of Imatinib and Wi-A, on ABL protein activity was studied by treating Imatinib as catalytic inhibitor and Wi-A as allosteric inhibitor (ABL-Imatinib_(cat)_-Wi-A_(allos)_ complex). Molecular docking and dynamic simulation studies for Imatinib-Wi-A combination revealed that Imatinib formed similar interactions at the catalytic site as compared to interactions formed in case of Imatinib-Asciminib combination (Table 2 and Supplementary File S1: Figures S2a,g). It still retained its activity as an ATP-competitive inhibitor of the ABL protein by forming majority of crucial interactions with ATP-binding residues (Table 2 and Supplementary File S1: Figures S1g and S2g). Imatinib induced an ‘out’ conformation of DFG motif and the ionic interaction between Lys290 and Glu305 was also maintained at the catalytic site of ABL-Imatinib_(cat)_-Wi-A_(allos)_ complex, as observed in the case of ABL-Imatinib_(cat)_-Asciminib_(allos)_ complex (Figure 3e). Wi-A interacted with the Asciminib interacting residues Arg351, Ala356, Glu481 and Leu529 (Supplementary File S1: Figures S2b, S2h, S3b and S3h), and induced same conformational changes at the allosteric site of the protein as observed in the case of Asciminib (Figure 3f). The MM/GBSA calculated binding energies of inhibitors in Imatinib-Wi-A combination was comparable to that of binding energies of inhibitors in Imatinib-Asciminib combination (Table 2). Also, the RMSDs and hydrogen bond profile of ABL-Imatinib_(cat)_-Wi-A_(allos)_ complex over 50 ns simulation trajectories were comparable to that of ABL-Imatinib_(cat)_-Asciminib_(allos)_ complex (Figure 2f,g). Altogether, combination of Imatinib-Wi-A was predicted to serve as inhibitor of ABL protein activity similar to Imatinib-Asciminib combination.

Based on the above data, Wi-A was expected to possess stronger anticancer activity than Wi-N. This was indeed endorsed by comparative cytotoxicity assays for Wi-A and Wi-N for several human cancer cell lines (Supplementary File S1: Table S2). Whereas Wi-A showed IC50 of ~1–3 μM, Wi-N showed IC50 of ~40–50 μM and was consistent with earlier reports [21,23,25,26,27,28,29,30,31].

## 4. Discussion

BCR-ABL regulates oncogenic signaling involving JAK/STAT, RAS-MEK, PI3K/AKT and FAK pathways, which prevent the cell from undergoing apoptosis. c-ABL binds to 14-3-3 and collects in the cytoplasm in its inactive form. Upon DNA damage, c-JUN phosphorylates 14-3-3 and releases the c-ABL protein. Free c-ABL contains three nuclear localization signals and one nuclear export signal in the carboxy-terminal region, which are mutually responsible for the cytoplasm to nucleus shuttling of c-ABL and vice versa [44,45]. In the nucleus, c-ABL binds to specific nuclear DNA domains to activate several genes. BCR-ABL as a result of the fusion gene formation remains constitutively active and without the help from c-JUN, it activates PI3K and MAPK as a tyrosine kinase to sustain survival and proliferation in the cells. Nuclear BCR-ABL is exported to the cytoplasm via direct interaction with the nuclear export receptor [46]. In the cytoplasm, thereafter, it is degraded by the ubiquitin-dependent proteasome pathway since the activated ABL is known to be more unstable than its inactive form [47]. This process is assisted by BAG1, which stimulates binding of BCR-ABL with 20S proteasome [48]. The relationship between the oncogenic tyrosine kinase BCR-ABL and the tumor suppressor p53 is a paradigmatic mechanism in aggressive cancers. A loss of p53 protein expression was observed as a result of BCR-ABL tyrosine kinase activity, followed by a delayed and deregulated differentiation of the multipotent myeloid cells [49], suggesting that p53 could also be regulated by BCR-ABL and contribute to multipotent cell development, differentiation and resistance to the extrinsic stimuli [50]. Stoklosa et al., (2004) showed that the BCR-ABL-transformed cells showed accumulation of p53 protein, similar to after ATR/ATM-dependent Ser15 phosphorylation of p53, in which the cells demonstrated a G_2_/M-cell cycle delay after stimulation of p21^WAF−1^ and genotoxic stress [50]. These effects were shown to suppress after p53 silencing, thus advocating for the role of BCR-ABL-p53 interaction in the drug resistance. Substantiating this, Abraham et al., (2016) showed that targeting p53 and c-MYC together could result into a synergistic death and activation of other essential mechanisms such as cell differentiation, selectively in the leukemic stem cells [51]. This mechanism was valid for both Imatinib responders and non-responders and was not evident by targeting BCR-ABL itself. Likewise, molecular targeting of BCR-ABL-led-mechanisms by molecules like Xanthohumol (NFκB and p53 modulator) [52], Oridonin (HSP70 modulator) [53], Forskolin (PP2A modulator) [54], BW18 (MAPK pathway inhibitor) [54], Taxodione (ROS inducer) [55] and Wogonin (STAT3/ NFκB inhibitor) [56] has been suggested.

The abnormally placed proto-oncogene *BCR-ABL1* (which codes for BCR-ABL protein) on chromosome 9 remains in a permanently activated state in leukemias. Characterized by these permanent aberrant mutations in the mature megakaryocytes, CML is known as one of the most aggressive diseases of uncontrolled proliferation. An evolution of treatment modalities has been discerned in the cases of CML, from Fowler’s solution (1865) to Imatinib (2000) and Ponatinib (2010) [57]; these could be a result of either significant scientific developments or failure of the treatment of choice. At present, Imatinib is the most commonly used drug in the CML clinics. Imatinib’s mechanism of action mainly revolves around the catalytic inhibition of the BCR-ABL protein, where it binds to the ATP-binding site and stabilizes its inactive state [11]. However, like many other synthetic molecules, the conventional tyrosine kinase inhibitors in monotherapy commonly produce severe adverse effects, chemo-resistance and thereafter disease recurrence [58], as a response to which an alternate tyrosine kinase inhibitor is prescribed that could also cause pharmacoeconomic and pharmacodynamic burden [59]. A variety of such combination strategies and their drawbacks have been mentioned previously [16,18,19,58]. Currently, the tyrosine kinase inhibitors in combination with interferon (Bosutinib/Dasatinib/Imatinib/Nilotinib, + pegylated Interferon-α), chemotherapeutic drugs (Imatinib, + Hydroxyurea/Cytarabine/Omacetaxine), immune modulators (Dasatinib, + Nivolumab/ Avelumab), PPAR-γ agonists (Thiazolidinediones), and DPPIV inhibitors (Gliptins) are under clinical trials [58]. CML disease relapse has been reported to be common after tyrosine kinase inhibitor therapy cessation [60]. Concomitantly activating apoptotic mechanisms and targeting BCR-ABL tyrosine kinase against the CML cells showed promise [60], by ensuring elimination of chronically phased CML stem/ progenitor cells. Alternate methods with curative intent such as allogenic bone marrow transplant or stem cell transplantation have also been tried, however, these are also limited with mismatches and several ineligibility criteria like age and comorbid conditions. Subsequently, the hunt for an adequate and satisfactory therapy against the CML that could also help to improve the overall quality of life remains obscure. This is where the natural molecules such as Wi-A gain importance for use, owing to their availability, affordability, tolerability, side-benefits, and broad-spectrum mechanisms of action.

Ashwagandha secondary metabolites Wi-A and Wi-N have been explored for their anti-cancer activities in several studies [28,29,32]. Previous studies have shown that Wi-A and Wi-N possess a multi-target activity by interacting with different proteins involved in cell growth, cell cycle regulation, metastasis and angiogenesis [28,29,31,33]. The ADME properties of these withanolides have been studied and reported elsewhere [23]. It has been shown that Wi-A and Wi-N agree Lipinski’s rule of five and have been predicted to have good oral absorption and good brain/blood partition coefficient [23]. Here, the in silico molecular docking and molecular dynamics simulation studies showed that Wi-A can serve as catalytic as well as allosteric inhibitor of ABL protein and mimics the interaction pattern of Imatinib at the catalytic site and Asciminib at the allosteric site. MM/GBSA binding energies of Wi-A in both catalytic and allosteric sites are better, compared to that of Wi-N, Imatinib and Asciminib. Therefore, it can be said that Wi-A has higher binding affinity to both catalytic and allosteric sites of ABL. As MM/GBSA binding energy calculation methods use implicit water models and do not account for entropy changes involved in ligand binding, they might differ from absolute values of actual binding affinities [41,61]. However, they can be used to evaluate the relative binding affinities of drug molecules to ABL [42].

The effect of the combination of Wi-A and Wi-N on ABL protein activity showed that Wi-A could serve as catalytic inhibitor and Wi-N as allosteric inhibitor of ABL protein. Wi-N induced same inactive conformation of ABL protein as induced by Asciminib and the natural substrate of ABL, i.e., Myr.

To estimate the simulation time needed to study the stable protein-ligand interaction, a 550 ns simulation was performed for one protein-ligand system (ABL-Wi-A_(cat)_-Wi-A_(allos)_ complex). From the RMSD plot of protein and ligand, it can be said that the protein and ligand attained a stable conformation within 50 ns of simulation (Supplementary File S1: Figure S4a). Average representative structure of protein-ligand complex from 0 to 50 ns was superimposed with the average representative structure from 50 to 500 ns (Supplementary File S1: Figure S4b). It was found that the protein structures and ligand orientations overlap with each other. Hence, it can be said that 50 ns simulation was good enough to sample the stable protein-ligand interaction in this case.

## 5. Conclusions

CML is a debilitating disease pathologically characterized by uncontrolled proliferation of the cancerous myeloid cells and molecularly characterized by the appearance of the Philadelphia chromosome. BCR and ABL are two distinct genes situated on separate chromosomes (22 and 9, respectively), which as a result of a reciprocal translocation mutation of ABL to 9th chromosome causes manifestation of fusion gene BCR-ABL. It produces BCR-ABL, a tyrosine kinase, via a constant ‘switched-on’ mode, which in turn is responsible for the activation of multiple cancer pathways. Currently, Imatinib and Asciminib are the standard treatments against the disease; but often suffer from drug resistance and post-therapy disease relapse. Using bioinformatics tools, we showed that the Ashwagandha-derived natural withanolides, Wi-A and Wi-N, could have potential to inhibit BCR-ABL driven oncogenic signaling. Wi-A interacted with the catalytic and allosteric sites of ABL stronger than Imatinib and Asciminib in combination. Wi-N interacted with the allosteric site of ABL and induced the same conformation as induced by Asciminib. Hence, the Ashwagandha derived secondary metabolites, Wi-A and Wi-N, are suggested as potential natural drug leads against cancer cells harboring ABL mutants. In vitro and in vivo preclinical and clinical studies on CML disease models are warranted to test the efficacy of Wi-A and Wi-N *per se,* and in combination with the conventional current clinical drugs used to treat this disease.

## Figures and Tables

**Table 1:** Binding energies obtained by separate docking of inhibitors at each site of ABL protein.

Inhibitors	ABL(Cat) (kCal/mol)	ABL(Allos) (kCal/mol)
Imatinib	−8.24	−3.60
Asciminib	−8.90	−6.16
Wi-A	−6.89	−3.58
Wi-N	−5.36	−2.67

**Table 2:** Binding energies and interactions formed by inhibitors at catalytic and allosteric site of ABL-Imatinib_(cat)_-Asciminib_(allos)_ complex (treated as control), ABL-Wi-A_(cat)_-Wi-N_(allos)_ complex, ABL-Wi-A_(cat)_-Wi-A_(allos)_ complex and ABL-Imatinib_(cat)_-Wi-A_(allos)_ complex. Interactions formed by Wi-A and Wi-N similar to our control inhibitors are highlighted in bold.

Complex	ABL-Imatinib_(cat)_-Asciminib_(allos)_		ABL-Wi-A_(cat)_-Wi-A_(allos)_		ABL-Wi-A_(cat)_-Wi-N_(allos)_		ABL-Imatinib_(cat)_-Wi-A_(allos)_	
Inhibitor	Imatinib_(cat)_	Asciminib_(allos)_	Wi-A(_cat)_	Wi-A_(allos)_	Wi-A_(cat)_	Wi-N_(allos)_	Imatinib_(cat)_	Wi-A_(allos)_
Docking Score (kCal/mol)	−8.24	−6.15	−6.89	−3.88	−6.89	−3.37	−8.24	−3.76
MM/GBSA Binding Energy before simulation	−74.55	−56.37	−14.88	−25.98	−14.88	−33.91	−74.55	−26
MM/GBSA Binding Energy during simulation	−78.11 ± 5.21	−54.00 ± 6.45	−82.19 ± 5.48	−67.00 ± 4.96	−75.87 ± 4.75	−42.11 ± 10.57	−79.10 ± 3.92	−57.65 ± 5.69
Hydrogen Bonds	Lys290 Thr338 Asn341	Glu481	Gly268 Glu305 **Thr338** Asp400	Gly188 Arg351 Ala356	Gly268 Asp400	Gln352 Val357	Met337 **Thr338**	Gly188 Ala356 Tyr454
Hydrophobic Interactions	Leu267 Tyr272 Val275 Val318 Phe336 Met337 Tyr339 Gly340 Cys388 Leu389 Ala399 Phe401	Ala356 Leu359 Leu360 Leu448 Ile451 Ala452 Tyr454 Pro480 Gly482 Cys483 Val487 Phe512 Ile521 Val525 Leu529	**Leu267** Gly268 **Tyr272** **Val275** Ala288 Ala306 Met309 **Val318** Ile332 **Phe336** **Met337** **Tyr339** **Gly340** **Leu389** **Ala399** **Phe401**	Gly188 Tyr158 Val190 **Ala356** Val357 **Tyr454** **Gly482** **Val525** **Leu529**	**Leu267** Gly268 **Tyr272** **Val275** Ala288 Val289 Met309 **Val318** Ile332 **Phe336** **Met337** **Gly340** **Leu389** **Ala399** **Phe401**	**Ala356** Val357 Val358 **Ile521** **Val525**	**Leu267** **Tyr272** **Val275** **Ala288** **Val318** **Phe336** **Met337** **Tyr339** **Gly340** **Leu389** **Phe401**	Tyr158 Gly188 Val190 Val354 **Ala356** Val357 **Leu359** **Tyr454** **Gly482** **Val525** **Leu529**
Positively charged interactions	His265 Lys290 Arg386	Arg351	**Lys290**	Arg185 Arg189 **Arg351**	**Lys290**		**Lys290**	Arg185 Arg189 **Arg351**
Negatively Charged Interactions	Glu335	Glu481	Glu305 **Glu335** Asp400	Glu157 Glu526 Glu528	**Glu335** Asp400	Glu353	**Glu335**	Glu157 **Glu481** Glu526
Polar Interactions	Thr334 Thr338 Asn341 Asn387 Ser284 Thr286 Thr338	Gln352	**Thr334** **Thr338** **Asn341**	Ser161 **Gln352** Asn355 Ser522	**Thr334** **Thr338** **Asn341**	Ser161 Ser162 **Gln352** Asn355 Ser522	**Thr334** **Thr338**	Ser161 **Asn355** **Ser522**

## Data Availability

The data generated during this study are included in this article.

## Supplementary Materials

The following are available online at https://www.mdpi.com/article/10.3390/biom12020212/s1, Supplementary File S1: Table S1: Inverse virtual screening docking score of Wi-A with protein kinases as target molecules. Supplementary File S1: Table S2: IC50 values for Wi-A and Wi-N against different human cell lines. Supplementary File S1: Figure S1: 2D interaction diagrams of inhibitors at catalytic and allosteric site of ABL. Interactions formed by (**a**) Imatinib at catalytic site and (**b**) Asciminib at allosteric site of ABL-Imatinib_(cat)_-Asciminib_(allos)_ complex. Interaction diagram of Wi-A at (**c**) catalytic site and (**d**) allosteric site of ABL-Wi-A_(cat)_-Wi-A_(allos)_ complex. Interactions formed by (**e**) Wi-A at catalytic site and (**f**) Wi-N at allosteric site of ABL-Wi-A_(cat)_-Wi-N_(allos)_ complex. Interaction diagram of (**g**) Imatinib at catalytic site and (**h**) Wi-A at allosteric site of ABL-Imatinib_(cat)_-Wi-A_(allos)_ complex. Supplementary File S1: Figure S2: Interactions fraction diagrams of inhibitors at catalytic and allosteric site of ABL. Interactions formed by (**a**) Imatinib at catalytic site and (**b**) Asciminib at allosteric site of ABL-Imatinib_(cat)_-Asciminib_(allos)_ complex. Interaction diagram of Wi-A at (**c**) catalytic site and (**d**) allosteric site of ABL-Wi-A_(cat)_-Wi-A_(allos)_ complex. Interactions formed by (**e**) Wi-A at catalytic site and (**f**) Wi-N at allosteric site of ABL-Wi-A_(cat)_-Wi-N_(allos)_ complex. Interaction diagram of (**g**) Imatinib at catalytic site and (**h**) Wi-A at allosteric site of ABL-Imatinib_(cat)_-Wi-A_(allos)_ complex. Vertical axis indicates the fraction of simulation time and interaction was maintained. Supplementary File S1: Figure S3: ABL residues at catalytic and allosteric sites interacting with inhibitors. Interactions formed by (**a**) Imatinib at catalytic site and (**b**) Asciminib at allosteric site of ABL-Imatinib_(cat)_-Asciminib_(allos)_ complex. Interaction diagram of Wi-A at (**c**) catalytic site and (**d**) allosteric site of ABL-Wi-A_(cat)_-Wi-A_(allos)_ complex. Interactions formed by (**e**) Wi-A at catalytic site and (**f**) Wi-N at allosteric site of ABL-Wi-A_(cat)_-Wi-N_(allos)_ complex. Interaction diagram of (**g**) Imatinib at catalytic site and (**h**) Wi-A at allosteric site of ABL-Imatinib_(cat)_-Wi-A_(allos)_ complex. Interactions that occur more than 30% of the simulation time are shown. Supplementary File S1: Figure S4: Results of 550 ns simulation of ABL-Wi-A_(cat)_-Wi-A_(allos)_ complex. (**a**) RMSD plot of Protein-ligand complex (**b**) Superimposed average representative structures of protein-ligand complexes from 0–50 ns of simulation and 50–550 ns of simulation. Wi-A molecules from 0–50 ns and 50–550 ns are shown in blue and magenta respectively. Supplementary File S2: Video of simulation trajectory: Imatinib at catalytic site and Asciminib at allosteric site of ABL-Imatinib_(cat)_-Asciminib_(allos)_ complex. Video is a sequence of 40 snapshots that were saved every 100 ps from 11–50 ns of simulation trajectories. Supplementary File S3: Video of simulation trajectory: Wi-A at catalytic site and allosteric site of ABL-Wi-A_(cat)_-Wi-A_(allos)_ complex. Video is a sequence of 40 snapshots that were saved every 100 ps from 11–50 ns of simulation trajectories. Supplementary File S4: Video of simulation trajectory: Wi-A at catalytic site and Wi-N at allosteric site of ABL-Wi-A_(cat)_-Wi-N_(allos)_ complex. Video is a sequence of 40 snapshots that were saved every 100 ps from 11–50 ns of simulation trajectories. Supplementary File S5: Video of simulation trajectory: Imatinib at catalytic site and Wi-A at allosteric site of ABL-Imatinib_(cat)_-Wi-A_(allos)_ complex. Video is a sequence of 40 snapshots that were saved every 100 ps from 11–50 ns of simulation trajectories.

## Abbreviations

CML: Chronic Myelogenous Leukemia; HPLC: High-Performance Liquid Chromatography; MTT: 3-(4,5-dimethylthiazol-2-yl)-2,5-diphenyltetrazolium bromide; RMSD: Root Mean Square Deviation; Wi-A: Withaferin-A; Wi-N: Withanone.
