# Supplementary material for: Computational Identification of BCR-ABL Oncogenic Signaling as a Candidate Target of Withaferin A and Withanone
Source: Biomolecules. 2022 Jan 26;12(2):212. doi: 10.3390/biom12020212 (PMC8961606; doi:10.3390/biom12020212)

Computational identification of BCR-ABL oncogenic signaling as a candidate target of Withaferin A and Withanone

Vidhi Malik[1], Navaneethan Radhakrishnan[1], Sunil C. Kaul[2], Renu Wadhwa[2] and Durai Sundar[1,3,]*

Supplementary File S1: Table S1. Inverse virtual screening docking score of Wi-A with protein kinases as target molecules.

Gene	Protein Name	Wi-A Docking Score (Kcal/mol)
ABL1	ABL proto-oncogene 1, non-receptor tyrosine kinase	-10.525
PRKG2	protein kinase, cGMP-dependent, type II	-9.453
PRKG1	protein kinase, cGMP-dependent, type I	-8.661
ERBB4	erb-b2 receptor tyrosine kinase 4	-8.661
PLK1	polo like kinase 1	-8.591
DAPK1	death associated protein kinase 1	-8.591
LCK	LCK proto-oncogene, Src family tyrosine kinase	-8.576
AKT1	AKT serine/threonine kinase 1	-8.556
MAP2K 1	mitogen-activated protein kinase kinase 1	-8.544
BTK	Bruton tyrosine kinase	-8.544
BRAF	B-Raf proto-oncogene, serine/threonine kinase	-8.416
ITK	IL2 inducible T-cell kinase	-8.145
SRC	SRC proto-oncogene, non-receptor tyrosine kinase	-8.143

Supplementary File S1: Table S2. IC50 values for Wi-A and Wi-N against different human cell lines.

Sl.	Cell line	Wi-A (in μM)	Wi-N (in μM)
1	MCF7	~ 2-3	~ 45-50
2	T47D	~ 2-3	~ 40-45
3	DLD1	~ 2-3	~ 45-50
4	HCT116	~ 1-2	~ 45-50
5	K562	~ 1-2	~ 40-45
6	HeLa	~ 2-3	~ 45-50
7	U2OS	~ 1-3	~ 45-50
8	T.Tn	~ 1-2	~ 40-45

Supplementary File S1: Figure S1. 2D interaction diagrams of inhibitors at catalytic and allosteric site of ABL. Interactions formed by **(a)** Imatinib at catalytic site and **(b)** Asciminib at allosteric site of ABL-Imatinib$_{(cat)}$-Asciminib$_{(allos)}$ complex. Interaction diagram of Wi-A at **(c)** catalytic site and **(d)** allosteric site of ABL-Wi-A$_{(cat)}$-Wi-A$_{(allos)}$ complex. Interactions formed by **(e)** Wi-A at catalytic site and **(f)** Wi-N at allosteric site of ABL-Wi-A$_{(cat)}$-Wi-N$_{(allos)}$ complex. Interaction diagram of **(g)** Imatinib at catalytic site and **(h)** Wi-A at allosteric site of ABL-Imatinib$_{(cat)}$-Wi-A$_{(allos)}$ complex.

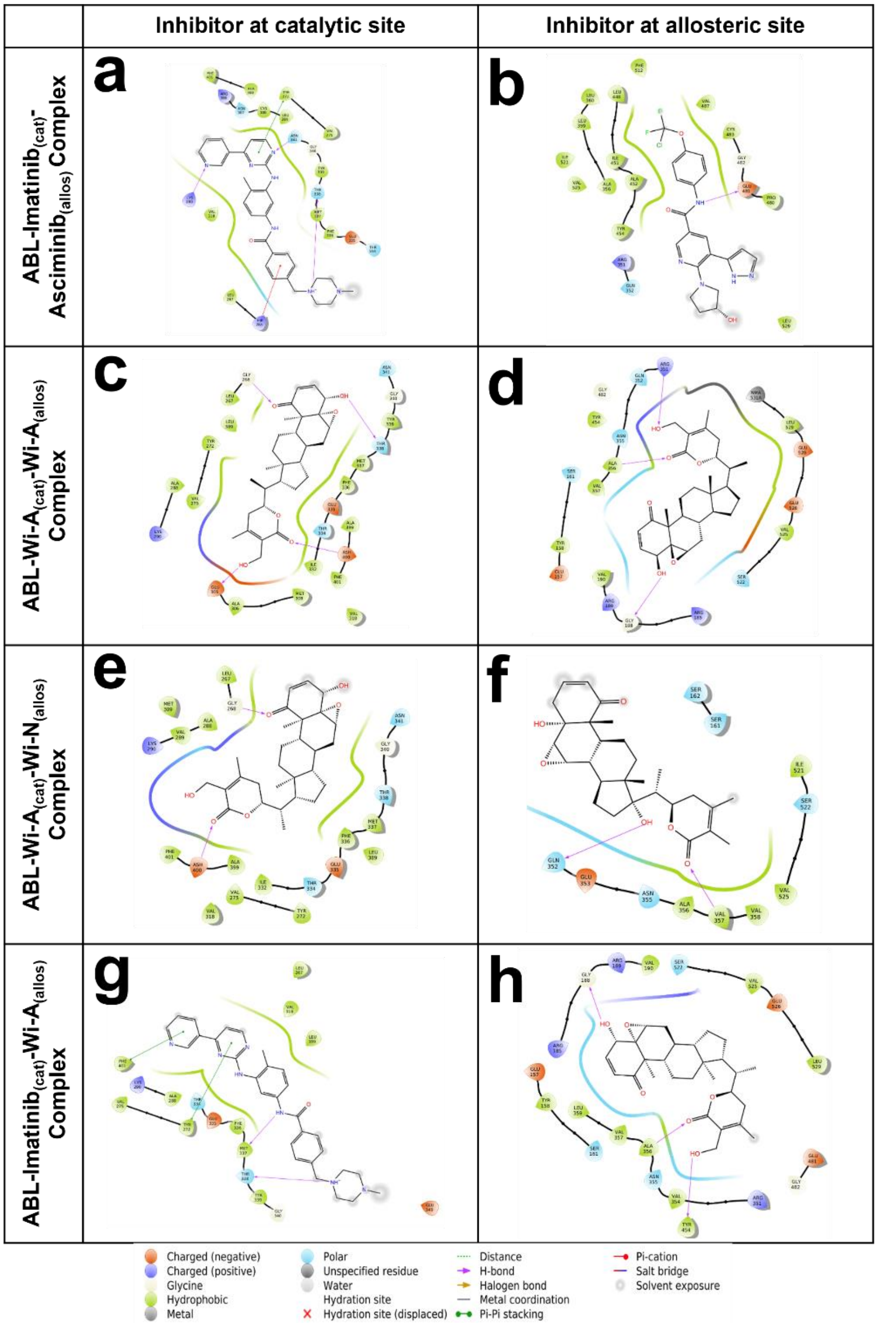

Supplementary File S1: Figure S2. Interactions fraction diagrams of inhibitors at catalytic and allosteric site of ABL. Interactions formed by **(a)** Imatinib at catalytic site and **(b)** Asciminib at allosteric site of ABL-Imatinib$_{(cat)}$-Asciminib$_{(allos)}$ complex. Interaction diagram of Wi-A at **(c)** catalytic site and **(d)** allosteric site of ABL-Wi-A$_{(cat)}$-Wi-A$_{(allos)}$ complex. Interactions formed by **(e)** Wi-A at catalytic site and **(f)** Wi-N at allosteric site of ABL-Wi-A$_{(cat)}$-Wi-N$_{(allos)}$ complex. Interaction diagram of **(g)** Imatinib at catalytic site and **(h)** Wi-A at allosteric site of ABL-Imatinib$_{(cat)}$-Wi-A$_{(allos)}$ complex. Vertical axis indicates the fraction of simulation time and interaction was maintained.

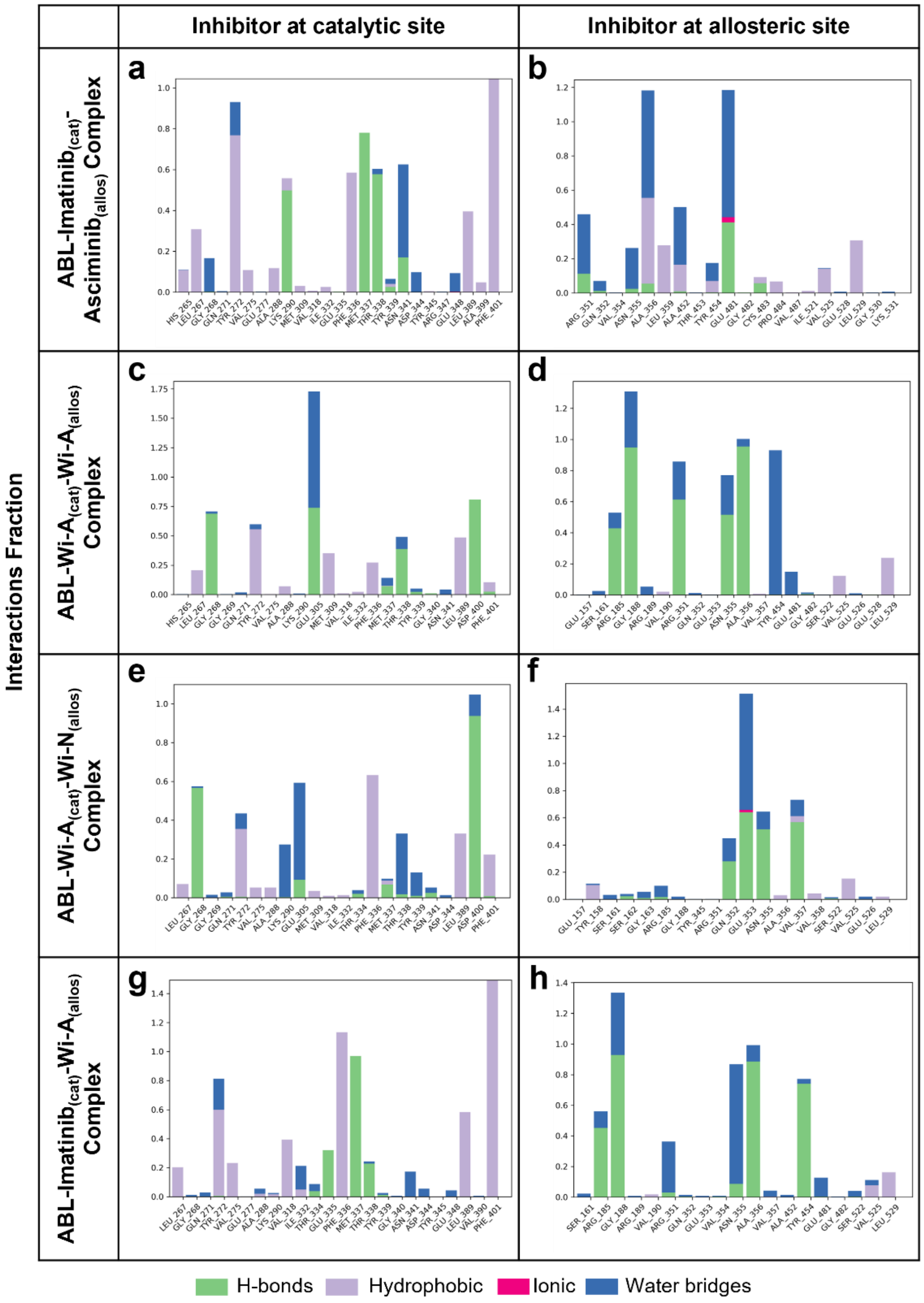

Supplementary File S1: Figure S3. ABL residues at catalytic and allosteric sites interacting with inhibitors. Interactions formed by **(a)** Imatinib at catalytic site and **(b)** Asciminib at allosteric site of ABL-Imatinib$_{(cat)}$-Asciminib$_{(allos)}$ complex. Interaction diagram of Wi-A at **(c)** catalytic site and **(d)** allosteric site of ABL-Wi-A$_{(cat)}$-Wi-A$_{(allos)}$ complex. Interactions formed by **(e)** Wi-A at catalytic site and **(f)** Wi-N at allosteric site of ABL-Wi-A$_{(cat)}$-Wi-N$_{(allos)}$ complex. Interaction diagram of **(g)** Imatinib at catalytic site and **(h)** Wi-A at allosteric site of ABL-Imatinib$_{(cat)}$-Wi-A$_{(allos)}$ complex. Interactions that occur more than 30%of the simulation time are shown.

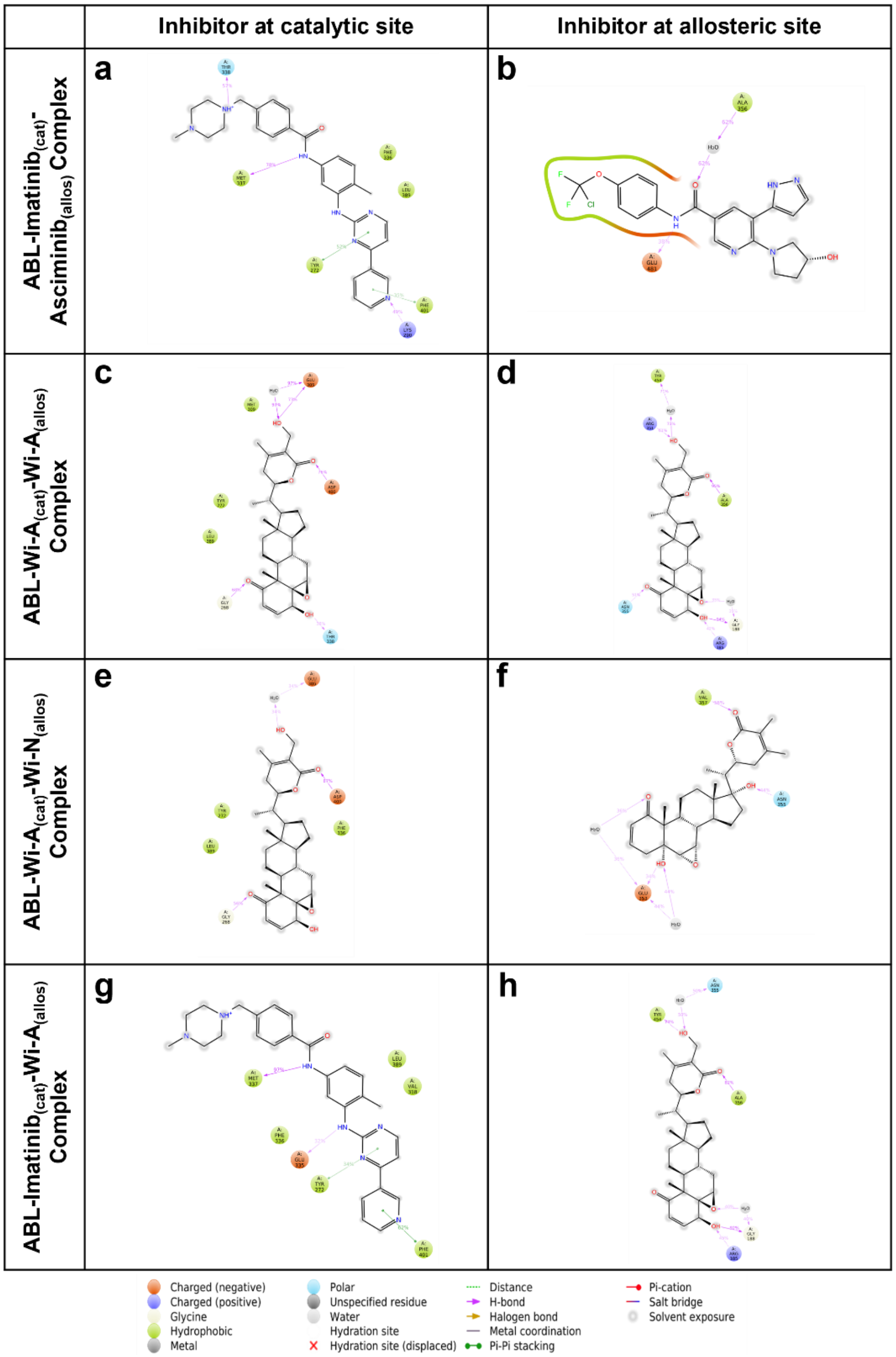

Supplementary File S1: Figure S4. Results of 550 ns simulation of ABL-Wi-A$_{(cat)}$-Wi-A$_{(allos)}$ complex. (a) RMSD plot of Protein-ligand complex **(b)** Superimposed average representative structures of protein-ligand complexes from 0-50 ns of simulation and 50-550 ns of simulation. Wi-A molecules from 0-50 ns and 50-550 ns are shown in blue and magenta respectively.

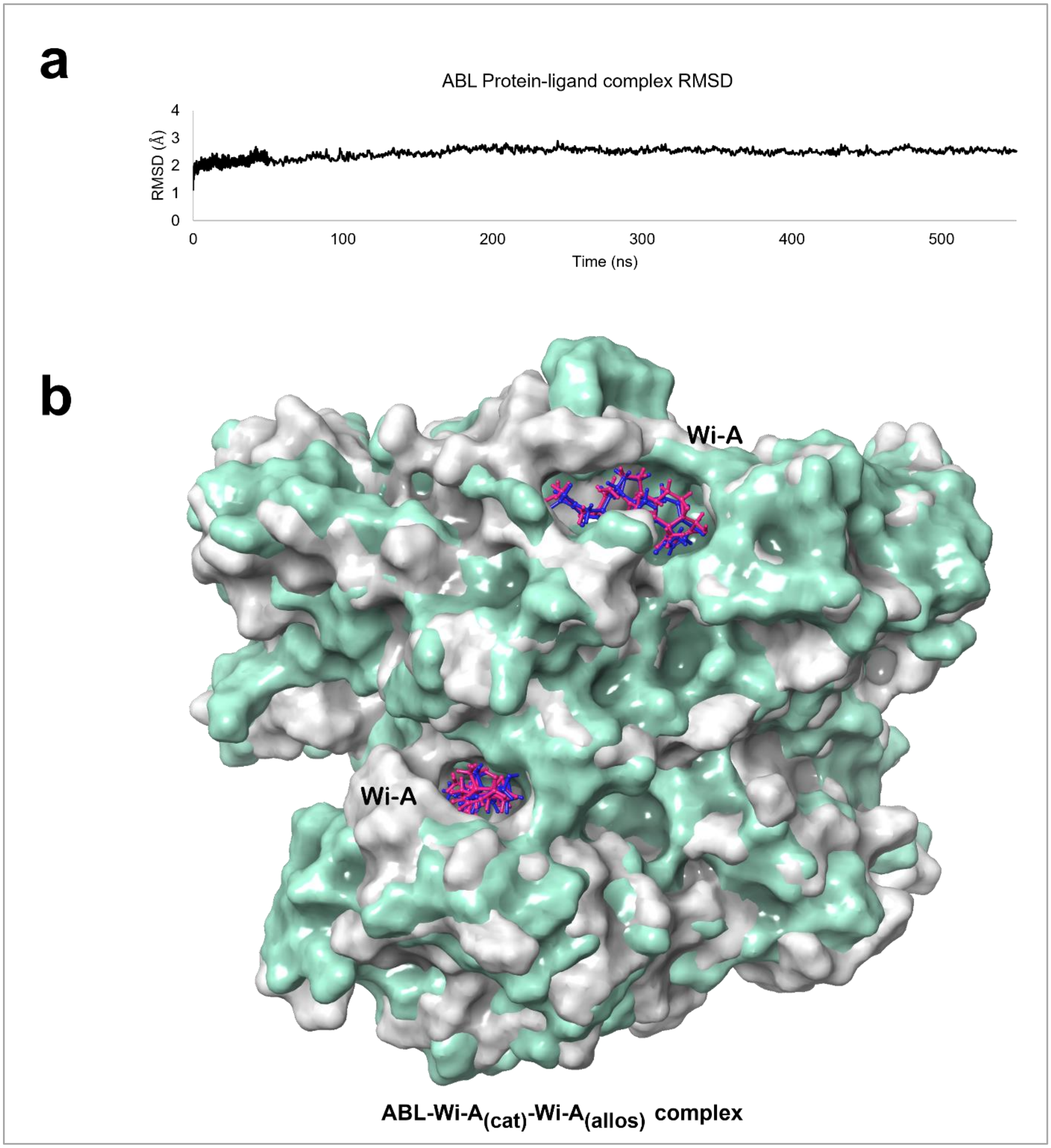

Supplement: Supplementary file 1 [file biomolecules-12-00212-s001.zip › 20220108-biomolecules-1423963-Suppl_File_S1.pdf]
